# The Efficacy of Two Intravenous Sedative Drugs in Management of Uncooperative Children for Dental Treatments

**Published:** 2015-03

**Authors:** Nasser Kaviani, Sanaz Ashrafi, Seyed Ebrahim Jabbarifar, Elham Ghaffari

**Affiliations:** 1 Dept. of Oral and Maxillofacial Surgery, Torabinejad Research Center, School of Dentistry, Isfahan University of Medical Sciences, Isfahan, Iran;; 2 Dentist, Graduated of Dental School, Dept. of Pediatric, School of Dentistry, Isfahan Branch (Khorasgan), Islamic Azad University, Isfahan, Iran;; 3 Pedodontist, Dept. of Pediatric Dentistry, School of Dentistry, Isfahan Branch (Khorasgan), Islamic Azad University, Isfahan, Iran;; 4 Postgraduate Student, Dept. of Pediatric Dentistry, School of Dentistry, Isfahan Branch (Khorasgan), Islamic Azad University, Isfahan, Iran;

**Keywords:** Intravenous Sedation, Midazolam, Ketamine, Fentanyl, Uncooperative Children

## Abstract

**Statement of the Problem:**

Some children do not show an appropriate cooperation with their dentist. A number of them cannot be managed by local anesthesia and the usual techniques used to control behaviors, so further steps are required to control their pain and anxiety. Pharmaceutical control is recommended through sedation or general anesthesia.

**Purpose:**

This study was aimed to evaluate two groups of drugs in intravenous sedation method.

**Materials and Method:**

In this clinical trial intervention study, patients were randomly divided into two groups of 18 and 20 and each group received either intravenous midazolam-ketamine or midazolam-fentanyl. During the procedure, 0.25mg midazolam was administered to both groups if needed. The scores of intraoperative sedation and operation conditions were evaluated and recorded by dental sedation teacher groups (DSTG) system in the 10^th^, 20^th^, 30^th^ and 40^th^ minutes of the operation. The results were analyzed by SPSS (version 16) using independent T-test, Wilcoxon, Mann-Whitney and Pearson Chi-Square tests as appropriated.

**Results:**

There was no significant difference between the two groups in sedation period (*p*= 0.55), recovery time (*p*= 0.18), Frankl score (*p*= 0.83**(**, score of intraoperative sedation and operating conditions (*p*> 0.05), and sedation complications (*p*= 0.612). In addition, no complication occurred in recovery.

**Conclusion:**

There was no significant difference between the two drug groups; both were appropriate in controlling children’s behavior.

## Introduction

Dental treatments seem painful to most children.[1] In many cases, this might cause the children to avoid necessary dental treatments or to tolerate it with great fear.[2-3] Eliminating negative memories of dentistry is important for children and is possible through sedation, and it induces positive attitude towards treatment with medications and other different methods. In this case, depending on the drug type and depth of sedation, the patient forgets a part or all of the treatment and nothing will be remembered after the treatment.

One of the methods to manage children in dental procedures is conscious sedation, during which the child would be sedated by use of sedative or anesthetic drugs, but is still able to cooperate with the dentist and respond to verbal commands. The patient’s comfort throughout the procedure and forgetting the events are among the characteristics of sedation. Using midazolam in dentistry is very acceptable and common and its forgetting effects are recognized in the literature.[4-5]

Three most commonly used sedation roots are inhalational, oral and intravenous. Although particular dosages of drug are recommended for conscious sedation in the mentioned techniques, usually none of these methods would cause an unarousable sleep, the reason they are named as conscious sedation.[6-7]

In intravenous method, the drugs that are used for conscious sedation are directly administered to venous blood through the veins. Conscious sedation causes the patient not to remember much of what happened during the procedure and after it is done.[8]

Benzodiazepines, ketamine, opioids, and propofol are the medications used in intravenous sedation. Based on the study by Nadin *et al.* (1997), midazolam caused anterograde amnesia in most patients.[9] A study by Azevedo *et al.* (2013) proved midazolam (in the studied dosage) to be an effective and innocuous medication for pediatric sedation.[10]

Ketamine is a non-barbiturate drug derived from phencyclidine;[11] it may be necessary to be combined with benzodiazepines in order to restrict the arousing reactions as well as enhancing the amnesia. Ketamine can result in significant but transient increase of systemic blood pressure, heart rate, and cardiac output through mediated stimulation of central sympathetic. Such effects can be adjusted by concurrent administration of benzodiazepines, opioids, or inhalational anesthetics.[6, 8, 12-13]

Okamoto *et al.* (1992) found that ketamine dosage would considerably decrease when used in combination with benzodiazepine.[14] Damle *et al.* (2002) enrolled a study concerning the efficacy and immunity of midazolam and propofol as sedating agents in managing uncooperative children and found that both are effective in sedation and have the least side effects.[15] According to a study by Golpayegani *et al.* (2012), combination of midazolam-ketamine provides sufficient sedative effect at lower dosage.[16]

Combination of several medications is used to improve the sedation quality. Midazolam as a frequently-used sedative drug might provide a better sedation in combination with ketamine (as an anesthetic drug with analgesic effect) and fentanyl (as an opioid sedative drug). A question would arise whether fentanyl or ketamine can provide better sedative effect when used in combination with midazolam. This study was carried out to compare the sedative properties and operating conditions of intravenous midazolam-fentanyl with midazolam-ketamine. 

## Materials and Method

This double-blind clinical trial was conducted on 38 healthy children aged 4-9, selected randomly from those referred to Department of Hospital Dentistry in Isfahan School of Dentistry. They were randomly allocated into two groups of 18 and 20; the patients in the first group were administered intravenous midazolam-ketamine while the second group received intravenous midazolam-fentanyl.

The inclusion criteria were healthy children with 4-9 years of age who referred to dental operating room and needed dental treatment on left upper teeth, with negative medical history of allergy, hepatic failure, renal failure, heart problems, chronic pulmonary disease, seizures, asthma, and psychological problems. The preoperative consultation was assessed and those who met the inclusion criteria were given similar preoperative instructions, and informed consents were obtained from parents. The instructions included of being fasted prior to the operation, not having taken sedative/hypnotic/ analgesic drugs, and having someone to accompany them on the day of operation.

On the operation day, the anesthesiologist randomly allocated the patients in one of the two groups, gave them an injection of the required drugs with respect to their weight, and managed the patient throughout the operation. Neither the dentist nor the person who was collecting data had a clue about the grouping method. 

After completing the sampling, the related codes were removed. The first group received intravenous midazolam (0.05mg/kg) and ketamine (0.5mg/kg), the second group intravenous midazolam (0.05mg/kg) and fentanyl (0.5µg/kg), both within a 2-minute time interval. 

Throughout the operation, midazolam (0.25mg) was administered to both groups if needed and the efficacy of both drug groups was evaluated in managing the children during the dental operation and after it. In addition, the side effects of the drugs were checked including restlessness, fear of injection, nausea and vomiting, complications of sedation and recovery, sedation period, and recovery period. Dental sedation teacher groups (DSTG) system[17] was used to assess the scores of intraoperative sedation .The operating condition in each 10-minute intervals from the beginning of operation (10, 20, 30, and 40 minutes from the start), as well as the data obtained through Frankl behavioral rating scale[18] concerning the ability to complete the dental treatment were all recorded on a sheet.

After the operation was over, the patient was subjected to standard care in recovery and was released when meeting the discharge conditions, and received comprehensive instructions thereafter. Throughout the operation, the patient was supervised by anesthesiologist and the vital signs and oxygen saturation was checked by using a pulse oximeter. The dentist administered local anesthesia according to the pediatric dentistry protocol. The results were analyzed by SPSS (version 16) using independent T-test, Wilcoxon, Mann-Whitney, and Pearson Chi-square tests as appropriated. 

## Results

**Figure 1:**
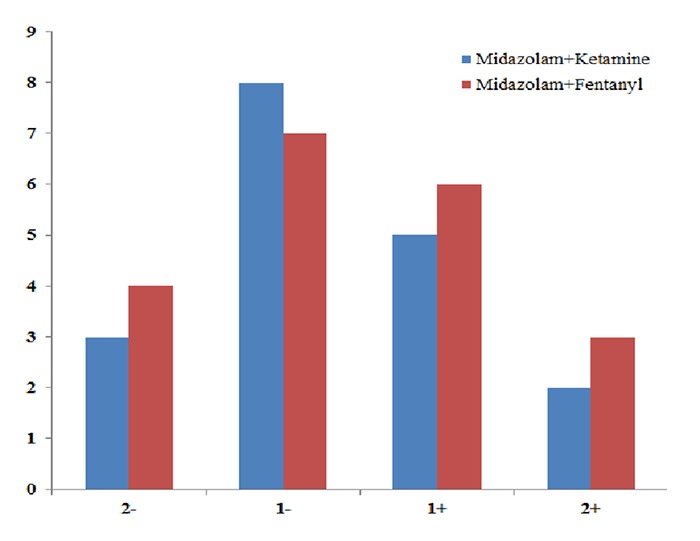
Frequency distribution of Frankl scores of the two studied groups

In this study, 38 patients were evaluated in two groups; 18 in midazolam-ketamine group and 20 in midazolam-fentanyl group. In midazolam-ketamine group, there were 8 boys (44.4%) and 10 girls (55.6%); in midazolam-fentanyl group, 12 were boys (60%) and 8 were girls (40%). Pearson’s Chi-square revealed no significant difference between the two groups concerning the gender (*p*= 0.338). The mean age in midazolam-ketamine group was 6.27 and in midazolam-fentanyl group, it was 6.75. Frequency distribution of -2 Frankl score was 16.7% in midazolam-ketamine group and 20% in midazolam-fentanyl group. Mann-Whitney test showed no significant difference between the drug groups concerning Frankl score during the dental treatment (*p*= 0.83) (Figure 1).

The mean recovery period and sedation time in midazolam-ketamine group were respectively 28.61 and 37.22 minutes, and in midazolam-fentanyl group, these numbers were 30 and 34.25 minutes, respectively. Independent T-test demonstrated that there was no considerable difference between the two groups concerning mean age, recovery, and sedation period (*p*> 0.05). One out of 18 patients in midazolam-ketamine group and 2 out of 20 patients in midazolam-fentanyl group developed sedation complications. Pearson’s chi-square test showed no significant difference between the groups concerning the sedation complications (*p*= 0.612). The mean score of intraoperative sedation in the 10^th^ minute was 2.27 in midazolam-ketamine group and 2.55 in midazolam-fentanyl group.

The mean score of operating conditions in the 10^th^ minute was 1.94 in midazolam-ketamine group and 1.85 in midazolam-fentanyl group (Table 1).

**Table 1:** Mean scores of intraoperative sedation and operating conditions in the two groups, divided based on time

**Time**	**Scores of intraoperative sedation**		**P value**	**Scores of operating conditions**		**P value**
	**Midazolam-fentanyl**	**Midazolam-ketamine**		**Midazolam-fentanyl**	**Midazolam-ketamine**	
	**Mean**	**Mean**		**Mean**	**Mean**	
10	2.277	2.55	0.27	1.94	1.85	0.64
20	1.941	2.277	0.12	2.44	2	0.1
30	1.846	1.916	0.72	2.60	2.40	0.6
40	1.727	1.727	1	1.60	1.66	0.75

Using Mann-Whitney test, no significant difference was detected between the two groups concerning the score of intraoperative sedation and the score of operating conditions in the 10^th^, 20^th^, 30^th^ and 40^th^ minutes (*p*> 0.05). Seventeen patients in midazolam-ketamine group and 18 in midazolam-fentanyl group got the intraoperative sedation score of 2 and 3 (ideal score) in the 10^th^ minute. This number in the 20^th^, 30^th^ and 40^th^ minutes of the operation was respectively 15, 11, and 8 in midazolam-ketamine group and 15, 10, and 8 in midazolam-fentanyl group (Table 2).

**Table 2:** Frequency distribution of score of intraoperative sedation in the 10^th^, 20^th^, 30^th^ and 40 minutes of the operation

**Score of** **intraoperative** **sedation**	** 10^th^ minute **				** 20^th^ minute **				** 30^th^ minute **				**40 minute**			
	**Midazolam-** **Fentanyl**		**Midazolam-** **Ketamine**		**Midazolam-** **Fentanyl**		**Midazolam-** **Ketamine**		**Midazolam-** **Fentanyl**		**Midazolam-** **Ketamine**		**Midazolam-** **Fentanyl**		**Midazolam-** **Ketamine**	
	**Frequency**	**%**	**Frequency**	**%**	**Frequency**	**%**	**Frequency**	**%**	**Frequency**	**%**	**Frequency**	**%**	**Frequency**	**%**	**Frequency**	**%**
1	1	5.6	0	0	2	11.1	2	10	2	11.1	2	10	3	16.7	3	15
2	11	61.1	11	55	14	77.8	10	50	11	61.1	9	45	8	44.4	8	40
3	6	33.3	7	35	1	5.6	5	25			1	5				
4			2	10			1	5								
5																

Fifteen patients in midazolam-ketamine group and 16 patients in midazolam-fentanyl group had the operating conditions score of 1 and 2 (the ideal score) at the 10^th^ minute. This number for the 20^th^, 30^th^, and the 40^th^ minutes of the operation was 9, 8, 10 individuals in midazolam-ketamine group and 14, 8, and 12 individuals in midazolam-fentanyl group (Table 3).

**Table 3:** Frequency of score of operating conditions in the 10^th^, 20^th^, 30^th^ and 40th minute of the procedure

**Score of ** **operating ** **conditions**	** 10^th^ minute **				** 20^th^ minute **				** 30^th^ minute **				**40 minute**			
	**Midazolam-** **Ketamine**		**Midazolam-** **Fentanyl**		**Midazolam-** **Ketamine**		**Midazolam-** **Fentanyl**		**Midazolam-** **Ketamine**		**Midazolam-** **Fentanyl**		**Midazolam-** **Ketamine**		**Midazolam-** **Fentanyl**	
	**Frequency**	**%**	**Frequency**	**%**	**Frequency**	**%**	**Frequency**	**%**	**Frequency**	**%**	**Frequency**	**%**	**Frequency**	**%**	**Frequency**	**%**
1	5	27.8	8	40	2	11.1	7	35	1	5.6	4	20	4	22.2	4	20
2	10	55.6	8	40	7	38.9	7	35	7	38.9	4	20	6	33.3	8	40
3	2	11.1	3	15	8	44.4	3	15	4	22.2	4	20				
4	1	5.6	1	5	1	5.6	2	10	3	16.7	3	15				

Wilcoxon test revealed significant difference between the score of sedation in the two groups of midazolam-ketamine and midazolam-fentanyl in the 10-20, 10-30 and 10-40 minutes of the operation (*p*< 0.05). There was also significant difference between the score of operating conditions in the two groups over the 10-20, 10-30, 20-40 and 30-40 minutes of the operation (*p*< 0.05). Neither group showed recovery complications, so no significant difference were found between the groups concerning the recovery complications.

## Discussion

Sedative drugs are used in pediatric dentistry to help managing the children behavior during the treatment procedure. They are expected to have the children change their behavior and take the sequential dental treatments with appropriate cooperation without using such medications.[19-21] In this study, the mean sedation time was 37.22 in midazolam-ketamine group and 34.25 minutes in midazolam-fentanyl group, with no significant difference between the groups. In midazolam-ketamine group, only one case and in the other group, two cases experienced hypoxia. Regarding the complications of sedation, neither group was superior nor was any recovery complication observed in either group.

In a study enrolled by Milnes *et al*. (2000), no complication was detected throughout the operation and recovery in children who received intravenous midazolam-nalbuphine-droperidol.[22] In the current study, during dental treatment in midazolam-ketamine group 3, 8, 5, 2 and in midazolam-fentanyl group 4, 7, 6, 3 individuals got Frankl score of -2, -1, +1, and +2, respectively; most children got -1 and the two groups had no significant difference in this regard.

In this study, no significant difference was found between the scores of intraoperative sedation in the two groups; indicating the effects of the two drug groups to be identical. This finding was due to the similarity in the types and effects of these medications, as well as complying with the recommended dosage. In each combination group of drugs, over 50% of the children had ideal sedation condition in the 10^th^, 20^th^ and 30^th^ minutes of the operation (sedation score: 2 and 3). Comparing the score of intraoperative sedation in midazolam-ketamine and midazolam-fentanyl group revealed a significant difference between the two groups in the 10-20, 10-30 and 10-40 minutes of operation. This might be because sedation functions better at the commencement of the operation. Somri *et al.* studied the sedation score of oral midazolam in three dosages of 0.5, 0.75 and 1 mg/kg and found that sedation score in 0.75 and 1mg/kg midazolam groups were higher than 0.5mg/kg group.[23]

Comparing the score of operating conditions, no significant difference was observed between midazolam-ketamine and midazolam-fentanyl groups; indicating the identical effect of both groups due to the similarity of type and effect of drugs, besides being used in recommended dosages. Sedative effects of these two drug combinations provided ideal operating conditions in the 10^th^ and 20^th^ minutes of the operation in more than 50% of children (operating condition score: 1 and 2). However, comparing the score of operating conditions in each group of midazolam-ketamine and midazolam-fentanyl revealed significant difference between the groups in the 10-20, 10-30, 20-40 and 30-40 minutes of the operation, which can be due to the better operating conditions provided at the beginning of the operation.

## Conclusion

There existed no significant difference between the two groups of midazolam-ketamine and midazolam-fentanyl in terms of sedation complications, recovery complications, Frankl score, sedation ,and recovery period, as well as score of intraoperative sedation, operating conditions during the 10^th^, 20^th^, 30^th^, and 40 minutes of operation, gender, and age. Both groups provided sufficient score of intraoperative sedation until the 30^th^ minute, and ideal operating conditions until the 20^th^ minute of the operation. Hence, until the 20^th^ minute of the procedure, drugs can be suitable for managing the children, neither was superior and each one was effective per se. Therefore, it seems that it makes no difference to combine midazolam with ketamine or fentanyl.

## References

[1] Berge TI (1999). Acceptance and side effects of nitrous oxide oxygen sedation for oral surgical procedures. Acta Odontol Scand.

[2] Koch G, Poulsen S (2001). Pediatric Dentistry.

[3] Sheller B (2004). Challenges of managing child behavior in the 21st century dental setting. Pediatr Dent.

[4] Rodrigo MR, Clark RN (1986). A study of intravenous sedation with diazepam and midazolam for dentistry in Hong Kong Chinese. Anaesth Intensive Care.

[5] Kupietzky A, Holan G, Shapira J (1996). Intranasal midazolam better at effecting amnesia after sedation than oral hydroxyzine: a pilot study. Pediatr Dent.

[6] Girdler NM (2009). Clinical sedation in dentistry.

[7] Boynes SG, Moore PA, Tan PM Jr, Zovko J (2010). Practice characteristics among dental anesthesia providers in the United States. Anesth Prog.

[8] Chandrachud W, Beltes C (2009). Intravenous conscious sedation. SAAD Dig.

[9] Nadin G, Coulthard P (1997). Memory and midazolam conscious sedation. Br Dent J.

[10] Azevedo ID, Ferreira MA, da Costa AP, Bosco VL, Moritz RD (2013). Efficacy and safety of midazolam for sedation in pediatric dentistry: a controlled clinical trial. J Dent Child (Chic).

[11] Panzer O, Moitra V, Sladen RN (2011). Pharmacology of sedative-analgesic agents: dexmedetomidine, remifentanil, ketamine, volatile anesthetics, and the role of peripheral muantagonists. Anesthesiol Clin.

[12] Craig DC, Wildsmith JA, Royal College of Anaesthetists (2007). Conscious sedation for dentistry: an update. Br Dent J.

[13] Skully M, Palmer D (2003). Consious sedation.

[14] Okamoto GU, Duperon DF, Jedrychowski JR (1992). Clinical evaluation of the effects of ketamine sedation on pediatric dental patients. J Clin Pediatr Dent.

[15] Arya VS, Damle SG (2002). Comparative evaluation of midazolam and propofol as intravenous sedative agents in the management of unco-operative children. J Indian Soc Pedod Prev Dent.

[16] Golpayegani MV, Dehghan F, Ansari G, Shayeghi S (2012). Comparison of oral Midazolam-Ketamine and Midazolam-Promethazine as sedative agents in pediatric dentistry. Dent Res J (Isfahan).

[17] Ransford NJ1, Manley MC, Lewis DA, Thompson SA, Wray LJ, Boyle CA (2010). Intranasal/intravenous sedation for the dental care of adults with severe disabilities: a multicentre prospective audit. Br Dent J.

[18] El Housseiny A, Farsi N, Alamoudi N, Bagher S, El Derwi D (2014). Assessment for the children's fear survey schedule-dental subscale. J Clin Pediatr Dent.

[19] McDonald DR, Avery D (2005). Pediatric Dentistry for the child and adolescent.

[20] Brosius KK, Bannister CF (2003). Midazolam premedication in children: a comparison of two oral dosage formulations on sedation score and plasma midazolam levels. Anesth Analg.

[21] Green SM, Rothrock SG, Lynch EL, Ho M, Harris T, Hestdalen R (1998). Intramuscular ketamine for pediatric sedation in the emergency department: safety profile in 1,022 cases. Ann Emerg Med.

[22] Milnes AR, Maupomé G, Cannon J (2000). Intravenous sedation in pediatric dentistry using midazolam, nalbuphine and droperidol. Pediatr Dent.

[23] Somri M, Parisinos CA, Kharouba J, Cherni N, Smidt A, Abu Ras (2012). Optimising the dose of oral midazolam sedation for dental procedures in children: a prospective, randomised, and controlled study. Int J Paediatr Dent.

